# Amygdala activation during unconscious visual processing of food

**DOI:** 10.1038/s41598-019-43733-2

**Published:** 2019-05-13

**Authors:** Wataru Sato, Takanori Kochiyama, Kazusa Minemoto, Reiko Sawada, Tohru Fushiki

**Affiliations:** 1 Kokoro Research Center, Kyoto University, 46 Shimoadachi, Sakyo, Kyoto 606-8501 Japan; 2 Brain Activity Imaging Center, ATR-Promotions, 2-2-2 Hikaridai, Seika, Soraku, Kyoto 619-0288 Japan; 3 Department of Neurodevelopmental Psychiatry, Habilitation and Rehabilitation, Graduate School of Medicine, Kyoto University, 53 Shogoin-Kawaharacho, Sakyo, Kyoto 606-8507 Japan; 4 Faculty of Agriculture, Ryukoku University, 1-5 Seta Oe-Cho Koya, Ohtsu, Shiga 520-2194 Japan

**Keywords:** Amygdala, Obesity

## Abstract

Hedonic or emotional responses to food have important positive and negative effects on human life. Behavioral studies have shown that hedonic responses to food images are elicited rapidly, even in the absence of conscious awareness of food. Although a number of previous neuroimaging studies investigated neural activity during conscious processing of food images, the neural mechanisms underlying unconscious food processing remain unknown. To investigate this issue, we measured neural activity using functional magnetic resonance imaging while participants viewed food and mosaic images presented subliminally and supraliminally. Conjunction analyses revealed that the bilateral amygdala was more strongly activated in response to food images than to mosaic images under both subliminal and supraliminal conditions. Interaction analyses revealed that the broad bilateral posterior regions, peaking at the posterior fusiform gyrus, were particularly active when participants viewed food versus mosaic images under the supraliminal compared with the subliminal condition. Dynamic causal modeling analyses supported the model in which the subcortical visual pathway from the pulvinar to the amygdala was modulated by food under the subliminal condition; in contrast, the model in which both subcortical and cortical (connecting the primary visual cortex, fusiform gyrus, and the amygdala) visual pathways were modulated by food received the most support under the supraliminal condition. These results suggest the possibility that unconscious hedonic responses to food may exert an effect through amygdala activation via the subcortical visual pathway.

## Introduction

Hedonic or emotional responses, such as experiences of pleasure, to food stimuli play critical roles in human life. Throughout evolutionary history, rapid hedonic reactions to food stimuli have helped humans acquire energy in environments where food resources were scarce. However, in today’s developed countries, where high-calorie food is abundant, reflexive hedonic responses to food increase the risk of overeating, obesity, and lifestyle-related diseases. Previous behavioral studies have shown that seeing and consuming food trigger hedonic responses, which, in turn, motivate food intake^1–7^.

A recent behavioral study has revealed that hedonic responses to food are elicited rapidly, even in the absence of conscious awareness of the food^8^. Using a subliminal affective priming paradigm^9^, the researchers found that subliminal presentation of food images increased preferences for subsequent face targets more so than subliminal presentation of mosaic images. These findings indicate that the unconscious hedonic responses elicited by the sight of food affect the hedonic evaluation of subsequent targets. Taken together, the behavioral findings suggest that hedonic responses to food are elicited both consciously and unconsciously.

Neuroimaging studies have investigated the neural mechanisms underlying the visual processing of food. Several previous functional magnetic resonance imaging (fMRI) studies investigated neural activity while food images were supraliminally presented. These studies consistently found that several brain regions, including the visual cortices (e.g., the posterior fusiform gyrus [FG])^10–15^ and the limbic regions (e.g., the amygdala)^10–12,16–19^ are more active during the presentation of food images than non-food images^20,21^. Based on these findings, some researchers proposed that the FG is responsible for the visual recognition of food images, which are then sent to the amygdala and several other related regions for hedonic processing^22^.

However, the neural mechanisms underpinning unconscious hedonic responses to food remain unknown. Although few studies have examined neural activation during the observation of subliminally presented food pictures, one fMRI study employed this paradigm and found activation in the left middle occipital gyrus, but not in the brain regions described above that are typically related to food processing^23^. This null finding is difficult to interpret because the study included multiple factors (e.g., testing healthy controls and individuals with anorexia nervosa) that might have overshadowed the food effects by increasing variance; moreover, null findings in general do not prove the lack of an effect^24^. Hence, questions about the extent to which the neural mechanisms involved in the conscious and unconscious visual processing of food differ remain unanswered. Several neuroimaging studies investigating the processing of emotional facial expression stimuli have reported that, as in the case of conscious food processing, unconscious processing of emotional facial expressions activates the amygdala^25–46^. Some lesion studies also reported crucial involvement of the amygdala in unconscious processing of emotional scenes^47,48^. Based on these data, we hypothesized that the amygdala would be active during both conscious and unconscious food processing.

Additionally, previous neuroimaging studies examining the processing of emotional expressions have reported differences in the information processing pathways used in conscious and unconscious emotional processing. Some studies found that emotional facial expressions are processed unconsciously through the subcortical visual pathway to the amygdala, which includes the superior colliculus and pulvinar^32,35,42,49^. Furthermore, the visual pathways involved in processing conscious and unconscious emotional facial expressions differ^50–52^. Based on these findings, we hypothesized that the visual pathways to the amygdala underpinning conscious and unconscious food processing would differ and that subcortical structures would be more strongly related to unconscious food processing.

**Figure 1:**
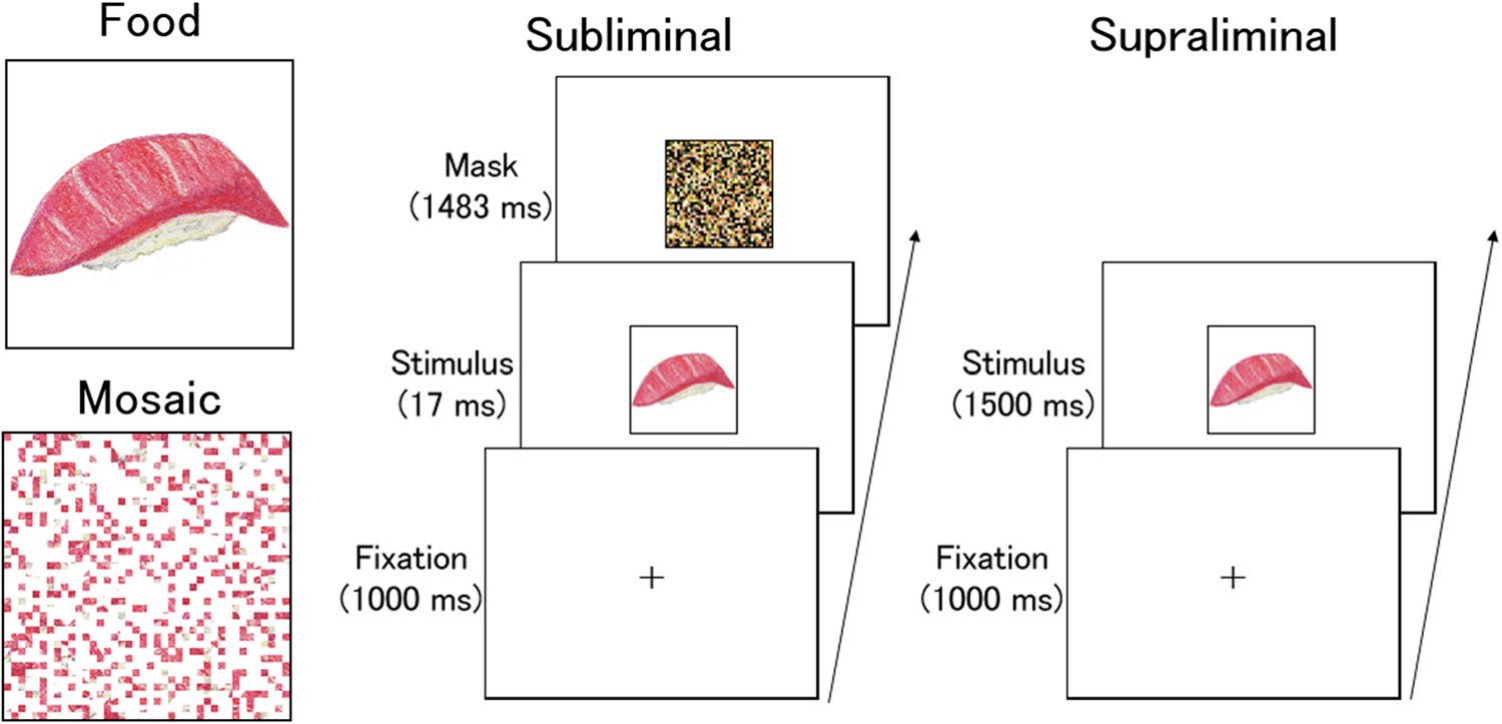
Schematic illustrations of stimuli (left) and trial sequences (right). Actual stimuli were photographs.

To test these hypotheses, we measured neural activity using fMRI while healthy participants (*n* = 22) observed food images presented supraliminally or subliminally (Fig. 1). As control stimuli for low-level visual features, such as luminance and color, mosaic images constructed from the food images were presented. We investigated the commonalities and differences in neural activity in response to food versus mosaic stimuli across presentation conditions. To test the generalizability of the effects across food categories, we used images of fast food and Japanese diet. Furthermore, we conducted dynamic causal modeling (DCM) analyses^53^ and compared models of the subcortical, cortical, and, dual visual pathways to the amygdala. After fMRI image acquisition, the stimuli were again presented to participants, as in a previous behavioral study^8^, and subliminal and supraliminal evaluation tasks were performed to investigate unconscious and conscious hedonic reactions to the food images.

## Results

The present study conducted random-effects analyses to identify significantly activated voxels at the population level^54^. The contrast images of neural activity during the viewing of food versus mosaic stimuli were entered into a within-subject one-factor analyses of variance (ANOVA) model with presentation condition (subliminal versus supraliminal) as a factor. Voxels were considered to be significantly activated if they reached the extent threshold of *p* < 0.05 (corrected for multiple comparisons) with a cluster-forming threshold of *p* < 0.001 (uncorrected).

### Neural activity under each presentation condition

Initially, the simple main effect of stimulus type (food versus mosaic) was tested under each presentation condition (Table 1). Under the subliminal condition, significant activity was detected only in the left amygdala and left occipital cortex. Under the supraliminal condition, significantly greater activation in response to food versus mosaic images was found in broad bilateral posterior regions, including the occipital, temporal, and parietal cortices, as well as in the amygdala. The activity peaks were located in the FG.

**Table 1:** Brain regions that exhibited significant activation in response to food versus mosaic images under each presentation condition.

Presentation	Area	Region	BA	Coordinates			*T*-value	Cluster Size	ROI
				*x*	*y*	*z*		(voxel)	
Subliminal	L. limbic	Amygdala	—	−28	−8	−16	3.5	4	*
	L. occipital	Middle occipital gyrus	19	−42	−82	30	4.5	286	
Supraliminal	L. occipital–temporal–parietal–limbic	Fusiform gyrus	37	−34	−50	−18	11.05	5948	
		Inferior occipital gyrus	19	−38	−74	−8	9.68		
		Middle occipital gyrus	18	−30	−92	12	8.74		
		Fusiform gyrus	20	−38	−24	−18	5.65		
		Hippocampus	—	−34	−14	−20	5.32		
		Superior parietal lobule	7	−24	−68	42	4.91		
		Amygdala	—	−30	−8	−18	3.84		
	R. occipital–temporal–parietal–limbic	Fusiform gyrus	37	32	−44	−18	10.49	5825	
		Fusiform gyrus	19	42	−64	−12	9.58		
		Inferior occipital gyrus	19	42	−86	0	8.26		
		Middle occipital gyrus	18	34	−82	8	7.02		
		Middle occipital gyrus	19	28	−72	32	5.24		
		Fusiform gyrus	20	40	−24	−18	4.75		
		Angular gyrus	7	28	−62	42	4.26		
		Amygdala	—	30	−4	−30	3.62		
		Hippocampus	—	22	−6	−18	3.59		

BA, Brodmann’s area; ROI, region of interest analysis; L, left; R, right.

### Commonalities in neural activity

**Figure 2:**
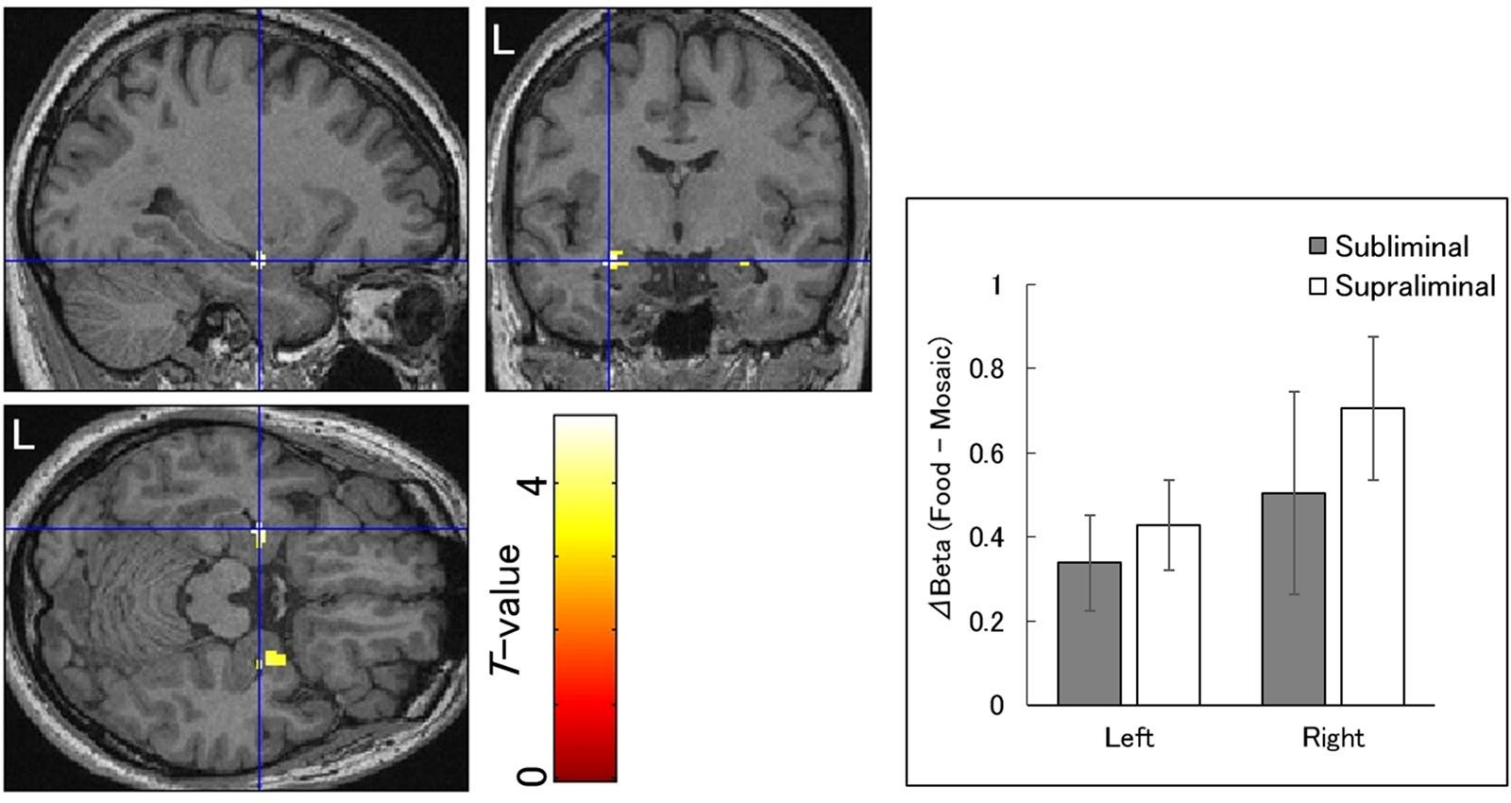
Statistical parametric maps indicating brain regions that were significantly activated in response to food versus mosaic images under the subliminal and supraliminal conditions. Areas of activation are rendered on the spatially normalized brain of a representative participant (left). The blue cross indicates the activation focus at the left amygdala, and the red–yellow color scale represents the *T*-value. Mean (±*SE*) beta value differences between the food and mosaic conditions (right). L, left hemisphere.

**Figure 3:**
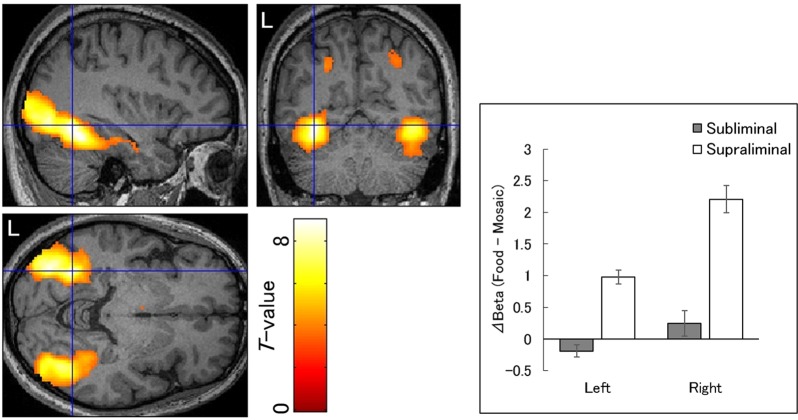
Statistical parametric maps indicating brain regions with significantly more activation in response to food versus mosaic images under the supraliminal condition compared with the subliminal condition. Areas of activation are rendered on the spatially normalized brain of a representative participant (left). The blue cross indicates the activation focus at the left fusiform gyrus, and the red–yellow color scale represents the *T*-value. Mean (±*SE*) beta value differences beteween the food and mosaic conditions (right). L, left hemisphere.

Next, a conjunction analysis was performed using interaction masking^55^ to test for commonalities in neural activity in response to food versus mosaic images across presentation conditions, as described previously^56,57^. The food images elicited more significant activation in the bilateral amygdala than the mosaic images under both the subliminal and supraliminal conditions (Table 2; Fig. 2, left). Beta value differences between the food and mosaic conditions in the amygdala foci in both hemispheres showed that the effects were positive under both the subliminal and supraliminal conditions (Hedges’ *g*_*av*_ = 0.60, 0.81, 0.43, and 0.84 for left subliminal, left supraliminal, right subliminal, and right supraliminal conditions, respectively; Fig. 2, right) relative to those in the FG (Fig. 3, right). Additional analyses with food category as a factor revealed no significant interactions of any other factors with this factor, indicating that the commonality effect in the amygdala was not qualified by food category.

**Table 2:** Brain regions that exhibited more significant activation in response to food versus mosaic images under both subliminal and supraliminal presentation conditions.

Area	Region	BA	Coordinates			*T*-value	Cluster Size	ROI
			*x*	*y*	*z*		(voxel)	
L. limbic	Amygdala	—	−30	−8	−18	4.73	24	*
R. limbic	Amygdala	—	24	−4	−20	4.01	28	*

BA, Brodmann’s area; ROI, region of interest analysis; L, left; R, right.

### Differences in neural activity

To test for differences in neural activity in response to food versus mosaic images across presentation conditions, interactions between stimulus type and presentation condition were analyzed. There was no significant activity in response to food versus mosaic images under the subliminal condition compared with the supraliminal condition (Table 3). There was significantly stronger activation in response to food than mosaic images under the supraliminal condition relative to the subliminal condition in broad bilateral posterior regions, including the occipital, temporal, and parietal cortices (Table 3; Fig. 3, left). The activation foci were detected in the bilateral FG. Beta value differences between the food and mosaic conditions in the FG foci in both hemispheres revealed large positive effects under the supraliminal, but not subliminal, conditions (Hedges’ *g*_*av*_ = −0.40, 1.78, 0.25, and 2.10 for left subliminal, left supraliminal, right subliminal, and right supraliminal conditions, respectively; Fig. 3, right). Additional analyses revealed no significant interactions between food category and any other factors, indicating that the posterior activity specific to the supraliminal condition was not qualified by food category.

**Table 3:** Brain regions that exhibited significant interaction activation in response to food versus mosaic images under specific presentation condition.

Presentation	Area	Region	BA	Coordinates			*T*-value	Cluster Size
				*x*	*y*	*z*		(voxel)
Subliminal	none							
Supraliminal	L. occipital–temporal–parietal	Fusiform gyrus	37	−34	−50	−8	9.18	4254
		Inferior occipital gyrus	19	−40	−70	−10	8.73	
		Middle occipital gyrus	18	−30	−92	12	7.41	
		Superior parietal lobule	7	−24	−66	42	4.64	
		Fusiform gyrus	20	−38	−24	−18	4.08	
		Middle occipital gyrus	19	−28	−78	26	3.61	
	R. occipital–temporal–parietal	Fusiform gyrus	37	34	−44	−18	8.49	4370
		Inferior occipital gyrus	19	40	−84	−2	6.96	
		Middle occipital gyrus	18	36	−84	4	6.15	
		Middle occipital gyrus	19	28	−72	32	4.04	
		Superior parietal lobule	7	28	−56	42	4.03	
		Angular gyrus	40	30	−52	42	3.74	
	L. occipital–parietal	Superior patietal lobule	7	−24	−66	42	4.73	277
		Middle occipital gyrus	7	−26	−58	38	4.02	

BA, Brodmann’s area; L, left; R, right.

### DCM

**Figure 4:**
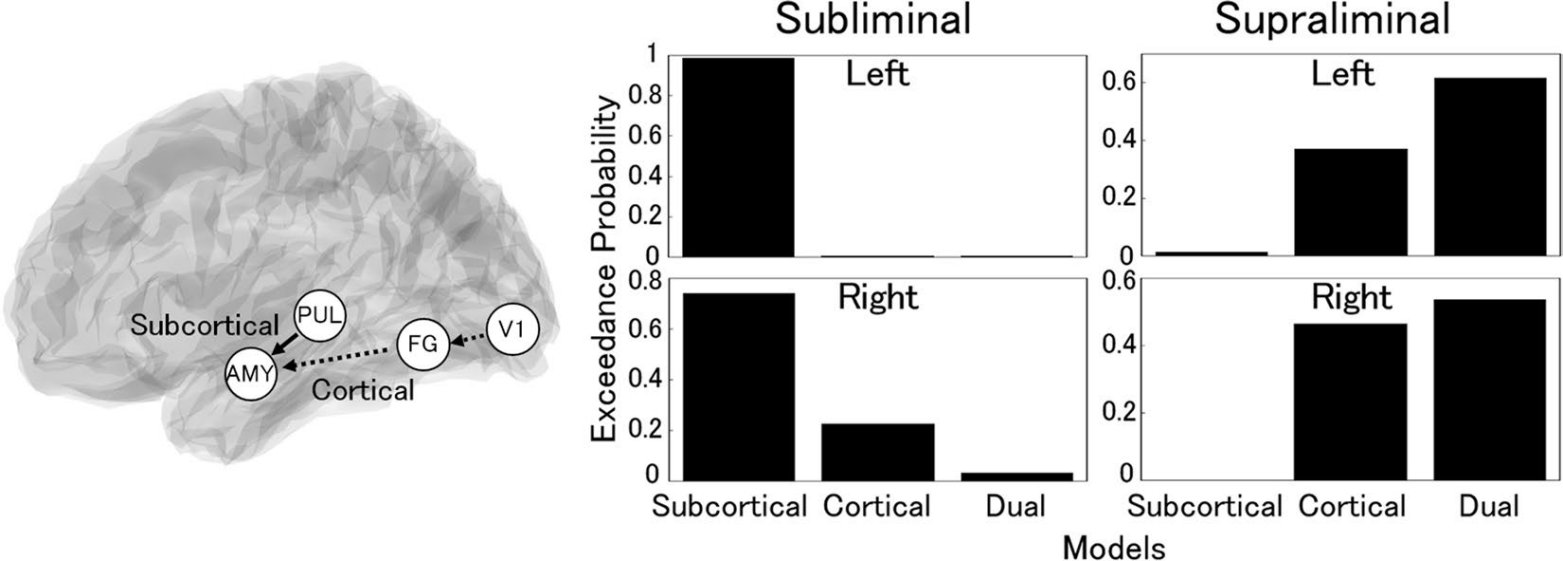
Models and model selection results of dynamic causal modeling. The analyzed brain regions of models are rendered on the spatially normalized brain (left). The arrows indicate connections between brain regions. Solid and dashed arrows indicate the subcortical and cortical pathway models, respectively. The dual pathway model contained both pathways. Exceedance probabilities of the models for the left and right hemispheres under the subliminal and supraliminal conditions are shown (right). AMY, amygdala; FG, fusiform gyrus; PUL, pulvinar; and V1, primary visual cortex.

DCM analyses were performed to identify the visual pathway to the amygdala in each hemisphere. The models in which the subcortical (pulvinar–amygdala), cortical (primary visual cortex [V1]–FG–amygdala), and dual visual pathways connected with the amygdala for processing food images were compared (Fig. 4). In both hemispheres, the exceedance probability of the random-effects Bayesian model selection indicated that the subcortical pathway model was optimal under the subliminal condition; by contrast, the dual pathways model was optimal under the supraliminal condition (Fig. 4).

### Behavioral data

To evaluate unconscious and conscious emotional responses to food images, preference ratings after fMRI image acquisition under the subliminal and supraliminal conditions, respectively, were analyzed. For the subliminal evaluation task, a dependent *t*-test revealed no significant differences in preference ratings for faces after presentation of the food versus mosaic images (mean ± *SE* = 4.5 ± 0.1 and 4.7 ± 0.2, respectively; *t*(21) = 1.07, *p* > 0.1). For the supraliminal evaluation task, a dependent *t*-test revealed a significant difference between the preferences for food versus mosaic images (mean ± *SE* = 6.9 ± 0.2 and 4.0 ± 0.3, respectively; *t*(21) = 8.55, *p* < 0.001). Binominal tests for the forced-choice discrimination task indicated that the participants’ discriminatory abilities did not significantly differ from chance levels (mean ± *SE* = 52.8 ± 1.9%; *p* > 0.2). Debriefing interviews confirmed that none of the participants consciously recognized food images during the subliminal fMRI image acquisition condition.

## Discussion

Our behavioral data for the supraliminal evaluation task demonstrated that the observation of food images was associated with higher preference ratings than the observation of mosaic images. This finding confirms considerable extant evidence showing that the observation of food induces positive emotional reactions^6,7^. The results of the subliminal evaluation task did not reveal any significant differences between the responses to food versus mosaic images, which is inconsistent with the results of a previous study^8^. This discrepancy may be due to methodological differences, such as the relatively shorter stimuli presentation times and the inclusion of fewer trials in the present study, which may have reduced the statistical power to detect behavioral effects. Because some previous fMRI studies also reported null effects in subliminal evaluation tasks despite amygdala activation in response to subliminally presented emotional stimuli^26,34^, it appears that the elucidation of unconscious emotional processing is more difficult on the basis of behavioral performance rather than fMRI signals.

More important, our cognitive conjunction analyses of the fMRI data revealed that the bilateral amygdala was more strongly activated in response to food than mosaic images under both the subliminal and supraliminal presentation conditions. This amygdala activation in response to supraliminally presented food images is consistent with the findings of a number of previous fMRI studies^20,21^. However, the present result differs from that of a recent study that reported a lack of amygdala activity in response to subliminally presented food images^23^. It is possible that methodological differences may account for the disparity in the results. For example, testing only healthy participants, investigating neural activity in hungry states, and a region of interest (ROI) approach for amygdala activity analyses were not employed in the previous study. Amygdala activation in response to subliminally presented food images may be compatible with previous neuroimaging findings of amygdala activation during the viewing of subliminally presented emotional facial expressions^31,32,34,44^, if it is assumed that images of food and facial expressions share the capacity to rapidly elicit emotional responses. The present findings showing similar amygdala activation patterns between the supraliminal and subliminal conditions are also consistent with the findings of previous studies that reported comparable amygdala activity in response to supraliminally and subliminally presented emotional expressions^25,28,42^. These data suggest that a majority of amygdala activity in response to emotional stimuli might occur during the early, unconscious stage. Consistent with this idea, a previous intracranial electroencephalography study reported that amygdala activity in response to emotional facial expressions exhibits a rapid onset, specifically after approximately 100 ms^58^. Furthermore, a previous fMRI study showed that amygdala activation reflects the hedonic value of food images^59^ and monkey lesion studies showed that amygdala damage impairs the ability to hedonically evaluate food^60–64^. Together with these data, the present results suggest that the amygdala is involved in both the unconscious and conscious hedonic processing of food.

Our interaction analysis revealed that broad posterior cortical regions were more strongly activated in response to supraliminally versus subliminally presented food images; activity peaked in the FG. The activation of the FG in response to supraliminally presented food images is consistent with several previous fMRI studies^20,21^ as well as fMRI findings demonstrating the involvement of the FG in the conscious perception of visual stimuli^51,65–67^. Taken together with these data, the present results suggest that activation of the FG is related to the conscious perception of food.

Furthermore, our DCM analysis revealed that the best models of the visual pathway to the amygdala were those including the subcortical pathway (pulvinar–amygdala) and the dual pathways (pulvinar–amygdala and V1–FG–amygdala) under the subliminal and supraliminal conditions, respectively. These findings suggest that the amygdala is activated by visual food stimuli via the subcortical visual pathway prior to the emergence of conscious awareness of food. Subsequently, the amygdala receives the processed visual signals of food via the cortical pathway. These results are consistent with anatomical evidence from diffusion tensor imaging studies showing white matter connections among the superior colliculus, pulvinar, and amygdala^68,69^. These results are also in line with those of previous fMRI studies showing that subcortical structures (pulvinar and amygdala) are activated and functionally coupled during the processing of unseen facial stimuli^32,35^. The results are also compatible with those of magnetoencephalography studies reporting that, in DCM analyses, early neural activity in response to emotional facial expressions (though not presented subliminally) was best explained by the model including the subcortical pulvinar–amygdala pathway^70,71^. Our model of the subcortical visual pathway to the amygdala under the subliminal condition is also consistent with data showing that the amygdala was activated in patients with damage to the entrance of the cortical visual areas^72–74^ and was functionally coupled with the pulvinar during the response to unseen emotional expressions^72^. In contrast to this literature on emotional processing, however, empirical and theoretical neuroscientific studies on food processing have not assumed involvement of the subcortical visual pathway to the amygdala^22^. The present results suggest that the hedonic responses to food are unconsciously accomplished by amygdala activation via the subcortical visual pathway.

Our results have several practical implications. First, because hedonic responses to food can lead to overeating^75^, which increases the risk of developing lifestyle-related diseases (e.g., type-2 diabetes)^76^, and because the control of overeating is generally difficult^77,78^, an understanding of the neuro-cognitive mechanisms underlying hedonic responses to food is imperative. In the context of this objective, our results suggest that the human brain has neural circuitry that unconsciously triggers hedonic responses to food stimuli independent of the circuits underlying conscious processing. Together with previous behavioral findings showing that unconscious emotional reactions are only minimally influenced by conscious reflection^9^ and neuroscientific findings showing that unconscious neural responses cannot be inhibited by the conscious will^79,80^, the present results suggest that the inhibition of unconscious hedonic responses to food using willpower is difficult. However, by understanding unconscious processes, we can consciously anticipate and indirectly modulate our responses^81^. For example, we can remove food stimuli in the environment to reduce hedonic eating, which is one of the basic procedures in cognitive behavioral therapy for eating control^82,83^. Additionally, it may be possible to develop cognitive procedures by addressing unconscious processes. For instance, a previous behavioral study found that a high working memory load reduced the impact of subliminally presented food images on subsequent behaviors^84^.

Second, our results revealed that the amygdala is involved in the unconscious hedonic processing of food. Because the amygdala is reportedly involved in various types of information processing other than food processing, amygdala activity in response to food may be indirectly modulated by non-food-related factors. For example, previous studies have shown that the amygdala is involved in certain aspects of social relationships, such as the perception of social support^85–87^; this suggests the possibility of social modulation of food processing. This idea may constitute a neural mechanistic explanation of evidence that therapies targeting social relationships (e.g., the Maudsley model of family therapy^88^) can improve eating behaviors^89,90^. We believe that understanding the neuro-cognitive mechanisms underlying unconscious hedonic responses to food could be helpful for developing effective interventions for healthy eating.

The present study also had several limitations. First, only mosaic images were presented as control stimuli and, thus, the types of information processing that could subliminally activate the amygdala remain unclear. The present study controlled for low-level visual properties of stimuli, such as brightness and color, because these variables reportedly modulate amygdala activity^91,92^ and food perception^93,94^. It is generally difficult to control low-level visual properties using different stimuli, such as objects^67,95,96^, and mosaic images have been used as control stimuli in several neuroimaging studies that investigated non-food processing^97,98^. However, because other types of non-emotional visual and cognitive factors might be important, further studies that employ different types of control stimuli to investigate unconscious amygdala activation in response to food will be necessary.

Second, although two different food categories were tested and no significant interactions with the other factors were found, the present investigation was not thorough in this regard and the findings should be considered as preliminary. The effect of food category is theoretically interesting, because previous fMRI studies have shown that the amygdala may preferentially process specific categories of emotional stimuli^74,99^ and behavioral studies found that the rapid perceptual and attentional processing of food images can differ depending on food ingredients^100–102^. Based on these data, it is possible that the amygdala is unconsciously and preferentially activated in response to specific food categories.

Third, only a small group of healthy participants was assessed in the present study. Because of the relatively small sample size, the present results cannot fully explain the null findings in other brain regions. Previous fMRI studies have reported activation in other brain regions in response to supraliminally presented food images^20,21^ and subliminally presented non-food stimuli^45,103^. These brain regions may possibly be associated with the unconscious hedonic processing of food images. As a related limitation, we focused on only a small number of regions with theoretical and empirical evidence for our DCM analyses. Because the amygdala receives projections from widespread subcortical and neocortical regions^104^, it may create functional couplings with more brain regions during food processing. Additionally, because the present participants were healthy volunteers, their neural responses may have differed from individuals with eating disorders. Previous behavioral studies have reported that individuals with anorexia nervosa exhibited greater reactivity to subliminally presented food images than healthy controls^105,106^, which suggests that there is a difference in the unconscious neural processing of food images in this population. Future studies with larger samples of healthy participants, as well as clinical populations, will further our understanding of the neural mechanisms underlying the unconscious hedonic processing of food.

Fourth, because the presentation conditions were not counterbalanced in the present study, there may have been confounding order effects. The order of the conditions was not changed because the supraliminal condition explicitly showed food images, which could have subsequently facilitated the conscious processing of food, and the primary objective of this study was the subliminal condition. However, preceding with the subliminal presentation of food images could have caused positive (e.g., priming) or negative (e.g., habituation) effects under the supraliminal condition. A more rigorous comparison between unconscious and conscious food processing will be an important matter for future studies.

Finally, there may be several factors that could modulate unconscious amygdala activity in response to food. For example, hunger level^10,12^ and body mass index (BMI)^107,108^ have been shown to modulate amygdala activity related to conscious food processing. These variables were measured and evaluated in our preliminary analyses and null effects were observed (see Methods). However, because the present sample was small and the range of these variables was restricted, the null findings were not conclusive. Several previous studies have suggested that other factors that were not measured in the present study, such as sleep history^109,110^, could also modulate amygdala activity in response to food. Additionally, it remains unclear whether amygdala activity in response to subliminal and supraliminal food images is tightly linked to emotional responses, because the emotional states of the participants during image acquisition were not assessed. Further investigation of the influences of these psychological and physiological variables on the modulation of rapid, unconscious neural food processing will be valuable.

In conclusion, our conjunction analyses revealed that the bilateral amygdala were activated more strongly in response to food images than mosaic images, under both the subliminal and supraliminal conditions. Interaction analyses revealed that broad bilateral posterior regions were specifically activated in response to food versus mosaic images, with peak activity seen in the FG, under the supraliminal condition compared with the subliminal condition. Under the subliminal condition, DCM analyses supported the model in which the subcortical visual pathway from the pulvinar to the amygdala was modulated by food. However, under the supraliminal condition, the analyses corroborated the model where both subcortical and cortical visual pathways (connecting the V1, FG, and amygdala) were modulated by food. These findings suggest the possibility that unconscious hedonic responses to food may exert effects through amygdala activation via the subcortical visual pathway.

## Methods

### Participants

The study included 22 healthy Japanese individuals (8 women and 14 men; mean ± *SD* age, 21.3 ± 1.6 years). All were in the normal weight range (mean ± *SD* BMI, 21.3 ± 3.1 kg/m^2^). Participants fasted for at least 3 h (mean ± *SD* 6.8 ± 3.9 h) before the experiment, which was conducted between 9:00 and 12:00. Their hunger level was assessed at the beginning of the experiment using a 5-point scale ranging from 1 (hungry) to 5 (satiated); the results showed that they were relatively hungry (mean ± *SD*, 2.1 ± 0.7). All participants were right-handed, as assessed by the Edinburgh Handedness Inventory^111^, and had normal or corrected-to-normal visual acuity. One additional volunteer participated, but his data were not analyzed because of his reported drowsiness. After the experimental procedures were fully explained, all participants provided informed consent for their participation. This study was approved by the Ethics Committee of the Medical Department, Kyoto University, and was conducted in accordance with the approved guidelines.

### Experimental design

The fMRI experiment used a within-subject two-factorial design, including presentation condition (subliminal, supraliminal) and stimulus type (food, mosaic). The food category (fast food, Japanese diet) factor was included in the exploratory analyses.

### Stimuli

Food stimuli (Fig. 1) were color photographs of fast food (three images in each of the four following subcategories: hamburgers, fried chicken, pizza, and doughnuts) and items from traditional Japanese diet (three images in each of the four following subcategories: sushi, roast fish, Japanese mixed rice, and noodles). The photographs were selected from images appearing on web sites and were cropped using Photoshop CS6 (Adobe, San Jose, CA, USA). The size of the stimuli was 4.9° vertically × 4.9° horizontally.

As control stimuli, we constructed mosaic stimuli from the food stimuli using MATLAB 6.5 (MathWorks, Natick, MA, USA). First, all food stimuli were divided into 40 × 40 small squares and reordered using a randomization algorithm. This rearrangement rendered each image unrecognizable as food. A mask stimulus was also prepared by creating a mosaic pattern consisting of fragments of food images not used in the experiment.

These stimuli were used in previous behavioral studies, and elicited appropriate subliminal and supraliminal hedonic responses to food versus mosaic images^8,112^. Specifically, the supraliminal images produced markedly higher liking ratings than those in response to mosaic stimuli (mean ± *SD* liking ratings of 7.2 ± 0.1, 7.0 ± 0.1, 3.6 ± 0.2, and 3.4 ± 0.2 for fast food, Japanese diet, fast food mosaic, and Japanese diet mosaic stimuli, respectively).

Face images were also used as target stimuli for the behavioral subliminal evaluation task. Grayscale photographs depicting neutral expressions displayed by 48 Japanese models (24 women and 24 men), which have been used in previous behavioral studies^8,112^, were employed. These stimuli were randomly assigned to the experimental conditions. The stimuli were 7.0° vertically × 7.0° horizontally in size.

### Presentation apparatus

The events were controlled by Presentation Software version 14.8 (Neurobehavioral Systems, Albany, CA, USA) implemented on a computer using Windows 7 (Microsoft, Redmond, WA, USA). The stimuli were projected from a liquid crystal projector (DLA-F110; Victor, Yokohama, Japan) at a refresh rate of 60 Hz to a mirror positioned in a scanner in front of the participants.

### Procedure

To determine the optimal stimulus presentation time under the subliminal condition, we conducted a preliminary threshold assessment experiment in the fMRI scanner using a procedure similar to that applied in a previous fMRI study^57^. We tested 10 participants (4 women and 6 men; mean ± SD age, 26.6 ± 3.3 years), none of whom took part in the subsequent fMRI experiment. The participants completed a total of 32 trials, 24 of which were similar to those under the subliminal condition during the fMRI experiment (i.e., presentation of a fixation point, a food image, and a mask at the center of the screen), except that the food images were presented for 17, 34, and 51 ms in each of eight trials. We also included eight trials with no food image (i.e., mask only) as the baseline condition. The order of trials was randomized. Participants were asked, “Did you see anything? If so, name it”. Participants first responded either “Yes” or “No”; in cases of a “Yes” response, they subsequently identified the stimulus. The mean ± *SE* percentages of “Yes (seen)” responses were 1.3 ± 1.4, 1.3 ± 1.4, 8.8 ± 2.9, and 12.5 ± 5.7 under the no-food, 17-, 34-, and 51-ms presentation conditions, respectively. Paired *t*-tests comparing the food and no-food conditions revealed significant differences only for the 34- and 51-ms presentation conditions (*t*(9) > 2.58, *p* < 0.05) in terms of the percentage of “Yes (seen)” responses. The mean ± *SE* percentages of correct responses were 0.0 ± 0.0, 3.8 ± 1.8, and 6.3 ± 3.0% under the 17-, 34-, and 51-ms presentation conditions, respectively. Based on these data, we presented food images for 17 ms under the subliminal condition in the subsequent fMRI experiment.

In the fMRI experiment, the participants completed a total of 256 trials, presented as two lots of 128 trials using a block design. Each run corresponded to one of the presentation conditions, and the order was fixed to the first subliminal and second supraliminal conditions. Each run consisted of 16 20-s task blocks interspersed with 16 20-s rest blocks (a blank screen). Each of the four stimulus conditions (fast food, Japanese food, fast food mosaic, and Japanese food mosaic) was presented in different task blocks within each scan. The order of task blocks within each run was pseudorandomized and the order of stimuli within each task block was randomized. Each task block consisted of eight trials. Instead of the usual stimulus, a red cross was pseudo-randomly presented as the target in eight trials placed throughout the task blocks. A break of approximately 1 min was inserted after the first run. Ten practice trials preceded the experimental trials.

At the beginning of each trial, a fixation point (i.e., a small black “+”) was presented for 500 ms at the center of the screen (Fig. 1). The stimulus was then presented at the same location. Under the subliminal condition, the stimulus was presented for 17 ms, followed by presentation of the mask in the same location for 1,483 ms. Under the supraliminal condition, the stimulus was presented for 1,500 ms, and no masking followed. Following stimulus presentation, a blank screen was displayed for 1,000 ms as an inter-trial interval. Participants were instructed to detect the red cross and to press a button with their right forefinger as rapidly as possible. These dummy tasks confirmed that participants were attending to the stimuli but did not require controlled cognitive, emotional, or behavioral processing of the stimuli. Performance on the dummy target-detection task was good (mean ± *SD* detection rate, 92.6 ± 12.7%; mean ± *SD* reaction times, 667.1 ± 109.8 ms).

As in a previous behavioral study using the same stimuli^8^, participants performed the subliminal and supraliminal evaluation and forced-choice discrimination tasks after MRI image acquisition.

Each of the subliminal and supraliminal evaluation tasks included a total of 48 trials involving preference judgments (24 food and 24 mosaic images). The order of conditions was randomized within each block. A short break was inserted after each block, and a longer break was inserted following completion of the subliminal condition. Participants initially participated in five practice trials to become familiar with the procedure under each presentation condition. In each trial of the subliminal evaluation task, a cross was initially presented at the center of the visual field for 1,000 ms as a fixation point. A priming food or mosaic image was then presented for 17 ms in either the left or the right visual field (the inside edge was 3.5° peripheral to the center); this was immediately followed by the presentation of a mask stimulus in the same location for 183 ms. The target face was then immediately presented in the central visual field for 1,000 ms. Finally, the rating display was presented until the participant entered a response. Participants were instructed to maintain their gaze at the location of the fixation cross throughout the trial. The participants’ task was to rate their preference for the target faces using a nine-point scale ranging from “extremely dislike” to “extremely like”^113^. They were asked to respond by pressing a key with the right index finger. In each trial of the supraliminal evaluation task, after a cross appeared for 1,000 ms as a fixation point at the center of the visual field, a target food or mosaic image was presented for 200 ms in either the left or the right visual field (the inside edge was 3.5° peripheral to the center). After a 1,000-ms presentation of a blank screen, the rating display appeared until the participant entered a response. Participants were instructed to maintain their gaze at the location where the fixation cross had appeared throughout the trial. Their task was to rate their preferences for the target food and mosaic images using the same nine-point scale used under the subliminal presentation condition.

A forced-choice discrimination task was administered after the preference ratings were completed. In total, 24 trials were conducted using food stimuli. In each trial of the discrimination task, a food stimulus was followed by a mask; this process was the same as the procedure under the fMRI subliminal condition. Then, two food stimuli, one of which had been presented, were presented in the upper and lower visual fields. The two stimuli were in the same food subcategory, and the participants indicated which food had been presented before. This task was based on the assumption that participants who had acquired a conscious awareness of the food stimulus would be able to select familiar stimulus based on low-level visual information.

An interview was subsequently conducted, and participants were asked whether they had consciously perceived the primes under the fMRI subliminal condition. Debriefing interviews were then conducted. After explaining the purpose of the experiment, we requested participants’ permission to analyze their responses under the subliminal condition; all participants consented to this request.

### MRI acquisition

Image scanning was performed on a 3-T scanning system (MAGNETOM Verio; Siemens, Malvern, PA, USA) using a 32-channel head coil. Elastic pads were used to stabilize participants’ head position. The functional images consisted of 40 consecutive slices parallel to the anterior–posterior commissure plane, covering the whole brain. A T2*-weighted gradient-echo echo-planar imaging sequence was used with the following parameters: repetition time (TR) = 2,500 ms; echo time (TE) = 30 ms; flip angle (FA) = 80°; field of view (FOV) = 192 × 192 mm; matrix size = 64 × 64; voxel size = 3 × 3 × 4 mm, without acceleration mode. The order of slices was ascending. After the acquisition of functional images, a T1-weighted high-resolution anatomical image was obtained using a magnetization-prepared rapid-acquisition gradient-echo sequence (TR = 2250 ms; TE = 3.06 ms; FA = 9°; inversion time = 1,000 ms; FOV = 256 × 256 mm; matrix size = 256 × 256; voxel size = 1 × 1 × 1 mm).

#### Image analysis: Preprocessing

Image and statistical analyses were performed using SPM12 (http://www.fil.ion.ucl.ac.uk/spm), implemented in MATLAB R2017b (MathWorks, Natick, MA, USA). Functional images of each run were realigned using the first scan as a reference to correct for head movements. We inspected the outputs of realignment, and confirmed that no participant required large (>2 mm) motion correction. Then, T1 anatomical images were coregistered to the first scan of the functional images. Following this, the coregistered T1 anatomical image was normalized to the Montreal Neurological Institute space using the unified segmentation-spatial normalization approach^114^. The parameters from this normalization process were then applied to each of the functional images, which appropriately correct for total intracranial volume differences^115^. Finally, these spatially normalized functional images were resampled to a voxel size of 2 × 2 × 2 and smoothed with an isotopic Gaussian kernel of 8-mm full-width at half-maximum to compensate for anatomical variability among participants.

#### Image analysis: Regional brain activity analysis

We used random-effects analyses to identify significantly activated voxels at the population level^54^. First, we performed a single-subject analysis^116^. Each condition (food, mosaic, or target) was embedded in a series of delta functions. The task-related regressor was modeled by convolving it with a canonical hemodynamic response function. We used a high-pass filter composed of a discrete cosine basis function with a cutoff period of 128 s to eliminate the artifactual low-frequency trend. Additionally, six realignment parameters were included as nuisance covariates to remove movement effects. Serial autocorrelation, assuming a first-order autoregressive model, was estimated from the pooled active voxels with a restricted maximum likelihood procedure and was used to whiten the data and the design matrix^117^. A contrast image of food versus mosaic images was generated for each presentation condition of each participant.

The contrast images for food versus mosaic stimuli were then entered into a within-subject one-factor ANOVA model with the factor of presentation condition (subliminal, supraliminal). This model allowed us to test the simple main effect of stimulus type under each presentation condition, the main effect of stimulus type, and the interactions between stimulus type and presentation condition.

Initially, the simple main effect of stimulus type (food versus mosaic) was tested under each presentation condition. Significantly activated voxels were identified if they reached the extent threshold of *p* < 0.05, corrected for multiple comparisons, with a cluster-forming threshold of *p* < 0.001 (uncorrected). A small volume correction^118^ with an anatomical mask was used for the amygdala, which was selected as the ROI based on our hypotheses. The search region in the amygdala was determined using the Anatomy Toolbox version 1.5^119,120^ by tracing the strict anatomical borders defined by the cytoarchitectonic map derived from data on human postmortem brains and by applying simple binalization (i.e., voxels in and out of the ROI were set to 1 and 0, respectively)^121^. Other areas were corrected for the entire brain volume.

As in previous studies^56,57^, we performed a conjunction analysis using interaction masking^55^ to test for commonalities in neural activity in response to food versus mosaic images across presentation conditions. For this analysis, we conducted a main-effect analysis of stimulus type (food versus mosaic) using *T*-statistics. To search for brain areas that showed similar activity across presentation conditions (supraliminal and subliminal), the main effect was exclusively masked by the *F*-test of the interaction between stimulus type and presentation condition. Thresholds were identical to those used in the aforementioned analysis of each presentation condition.

To test for differences in neural activity in response to food versus mosaic images across presentation conditions, interactions between stimulus type and presentation condition were analyzed. We analyzed the specific instances in which higher levels of activity were more strongly associated with one presentation condition than with another. Thresholds were identical to those used in the aforementioned analyses.

The analyses of commonalities and differences in neural activity were conducted a second time with the additional factor of food category to check the generalizability of the effects. We performed *F*-tests of interactions among stimulus type, presentation condition, and food category within the significant clusters obtained in the above analyses using the same thresholds.

Because some previous studies have suggested that age^122,123^, gender^124,125^, BMI^107,108^, and hunger level^10,12^ are potentially confounding factors for amygdala activity, we conducted preliminary analyses using the same one-factor ANOVA model as described above with presentation condition as a factor and age, gender, BMI, and hunger scores as covariates. The results revealed the same patterns of significant activity in the amygdala and FG. Furthermore, none of the covariates were significantly associated with amygdala activity. Therefore, we omitted these covariates from the reported analyses.

To visualize the activation patterns of the amygdala and FG, we calculated differences in beta values between the food and mosaic conditions at the activation foci in the conjunction and interaction analyses described above. We visualized the beta value differences in the figures and reported unbiased effect sizes, Hedges’s *g*_*av*_ (i.e., Cohen’s *d*_*av*_ applied Hedges’ correction)^126^. Additionally, to confirm that these activations were not derived from outliers, we conducted preliminary analyses using one-sample Wilcoxon signed rank tests. The results revealed that differences in beta values between the food and mosaic conditions for amygdala activity in both hemispheres under the subliminal and supraliminal conditions, and for FG activity in both hemispheres under the supraliminal condition, were significantly different from zero (*p* < 0.05).

The brain structures were anatomically labeled using Talairach Client (http://www.talairach.org/) and the Automated Anatomical Labelling atlas provided by the MRIcron software (http://www.mccauslandcenter.sc.edu/mricro/mricron/).

#### Image analysis: DCM analysis

We performed DCM analysis^53^ using DCM12 in the SPM12 software to investigate the effective connectivity between the amygdala and other brain regions. DCM allows for the modeling of three different types of interaction in a neural network: (1) the driving input, which represents the influences of exogenous input on neural states; (2) the fixed connections, which represent baseline (i.e., applicable to all experimental conditions) connectivity among neural states; and (3) the modulation of intrinsic connections by experimental manipulations. Based on our interests, as described in the Introduction, we investigated the modulatory effect of stimulus type (food versus mosaic).

To construct the driving and modulatory inputs in the current DCM analysis, we remodeled the single-subject analyses. The design matrix contained the following two experimental factor-specific regressors: the visual input (i.e., all food and mosaic stimuli) was the driving input in the DCM, and the stimulus type was the modulatory input. Target detection was included as an effect of no interest. Other nuisance regressors (realignment parameters and constant terms), high-pass filters, and serial autocorrelations were applied using the same settings described above for the regional brain activity analyses.

To investigate the visual pathway to the amygdala, four ROIs in the left and right hemispheres were selected: the pulvinar (left: *x* −20, *y* −28, *z* −2; right: *x* 22, *y* −28, *z* 0), V1 (left: *x* −16, *y* −94, *z* −14; right: *x* 14, *y* −92, *z* −14), FG (left: *x* −34, *y* −50, *z* −8; right: *x* 34, *y* −44, *z* −18), and amygdala (left: *x* −30, *y* −8, *z* −18; right: *x* 24, *y* −4, *z* −20). The coordinates of the pulvinar and V1 were derived from the activation foci in response to visual input within the search regions, which included spheres that were 8-mm in radius and centered on the activation foci identified in a previous study^127^ and Brodmann’s area 17 as defined by the WFU PickAtlas^128^, respectively, in the regional brain activity analysis at the group level, as described previously^129^. The coordinates of the FG and amygdala were also defined based on the results of the group-level interaction and commonality analyses, respectively. These ROIs were determined according to the hypothesis described in the Introduction. The first eigenvariate of all voxels within a 3-mm radius around the selected coordinate was extracted as ROI time series for each participant. The ROI time series were adjusted for the effects of no interest and nuisance regressors, high-pass filtered, and corrected for serial correlation.

Next, hypothetical models (cf. Fig. 4) were constructed for each hemisphere of each participant. For the subcortical pathway model, a driving input was set into the pulvinar, an intrinsic connection was prepared from the pulvinar to the amygdala, and the modulatory effect of stimulus type on this intrinsic connection was predicted. This subcortical network was constructed based on theoretical^52^ and empirical^32^ evidence regarding the processing of emotional facial expressions. Although the studies posited that the superior colliculus sends input to the pulvinar, we did not include the superior colliculus in the current model because this region is located adjacent to the pulvinar, making it difficult to distinguish using the defined ROI selection method. For the cortical pathway model, a driving input was set into the V1, the intrinsic connections were made from the V1 to the FG and from the FG to the amygdala, and the modulatory effect of stimulus type was modeled on both intrinsic connections. The dual pathway model contained all elements in both the subcortical and cortical models. To examine the visual pathways to the amygdala related to unconscious and conscious food processing, separate analyses were conducted under each presentation condition, and we identified the most appropriate model using random-effects Bayesian model selection^130^. We used the exceedance probabilities as the evaluation measures based on the assumption that one particular model was more likely to be accurate than any other model given the group data^131,132^.

### Behavioral data analysis

All behavioral data were analyzed using SPSS 16.0J software (SPSS Japan, Tokyo, Japan). The preference data were analyzed separately for each presentation condition using dependent *t*-tests. Data from the forced-choice discrimination task for each participant were analyzed using a binominal test. *P* values < 0.05 were considered to indicate statistical significance.
